# Case Report: Identification of the First Synonymous Variant of Myosin Binding Protein C3 (c.24A>C, p.P8P) Altering RNA Splicing in a Cardiomyopathy and Sudden Cardiac Death Case

**DOI:** 10.3389/fcvm.2022.806977

**Published:** 2022-03-02

**Authors:** Jie-Yuan Jin, Jiao Xiao, Yi Dong, Yue Sheng, Ya-Dong Guo, Rong Xiang

**Affiliations:** ^1^School of Life Sciences, Central South University, Changsha, China; ^2^Department of Forensic Science, School of Basic Medical Sciences, Central South University, Changsha, China; ^3^Hunan Key Laboratory of Animal Models for Human Diseases, School of Life Sciences, Central South University, Changsha, China; ^4^Hunan Key Laboratory of Medical Genetics, School of Life Sciences, Central South University, Changsha, China

**Keywords:** *MYBPC3*, sudden cardiac death, dilated cardiomyopathy, synonymous variant, splicing

## Abstract

**Background:**

Sudden cardiac death (SCD), based on sudden cardiac ejection cessation, is an unexpected death. Primary cardiomyopathies, including dilated cardiomyopathy (DCM), are one of main causes of SCD. The DCM is characterized by a cardiac dilatation and a reduced systolic function with a prevalence of 1/250 in adults. The DCM has been reported with more than 60 disease-causing genes, and *MYBPC3* variants are one of the most common and well-known causes of DCM.

**Methods:**

We identified a 29-year-old female who died of SCD. We performed a whole-exome sequencing (WES) to detect her genetic etiology and used minigene modeling and immunohistochemistry staining to verify the pathogenicity.

**Results:**

We determined that the woman died of SCD caused by DCM due to an identified novel synonymous variant of *MYBPC3* (NM_000256.3: c.24A>C, p.P8P) in the deceased. The variant can result in abnormal splicing, which was confirmed by minigene models and immunohistochemistry staining.

**Conclusion:**

We may have identified the first deleterious synonymous variant of *MYBPC3* in an SCD case and verified its significant impact on RNA splicing. Our description enriched the spectrum of *MYBPC3* variants and emphasized the significance of synonymous variants that are always disregarded in genetic screening.

## Introduction

Sudden cardiac death (SCD) is an unexpected death based on sudden cardiac ejection cessation. It accounts for 15–20% of unnatural death in developed countries (1, 2). The prominent symptoms of SCD include chest pain, dyspnea, palpitations, presyncope, and syncope. Primary electrical disorders, atherosclerosis, congenital cardiovascular diseases, and cardiomyopathies are the four main causes of SCD (3).

Cardiomyopathies can be divided into dilated cardiomyopathy (DCM), hypertrophic cardiomyopathy (HCM), restrictive cardiomyopathy (RCM), arrhythmogenic right ventricular cardiomyopathy (ARVC), and myocarditis (4). Among these cardiomyopathies, DCM is the most frequent, with a prevalence in adults of at least 1/250 (5). The DCM is characterized by cardiac dilatation and reduced systolic function. A heritable pattern is present in 20–35% of cases (6). Most of inherited DCMs show an autosomal dominant pattern and are usually present in the second or third decade of life (5).

Dilated cardiomyopathy (DCM) has been reported with more than 60 disease-causing genes, involving the sarcomere, Z disc, sarcoglycans, cytoskeletal complex, nuclear envelope, potassium and sodium channels, heat shock proteins, transcription factors, and mitochondria proteins (5).

The *MYBPC3* variants are one of the most common and well-known causes of DCM. The *MYBPC3* encodes the cardiac isoform of myosin-binding protein C and is located in the cross-bridge-bearing zone of A bands in striated muscle (7). An *MYBPC3* defect would result in a striking pattern of sarcomere disorganization and dysgenesis, triggering cardiomyopathy (8).

In the study, we identified a 29-year-old female who died of SCD caused by DCM. We detected a synonymous variant of *MYBPC3* (NM_000256.3: c.24A>C, p.P8P) in the deceased. The variant occurred in the penultimate base of exon 1 and resulted in abnormal splicing. Minigene and immunohistochemistry verified its pathogenicity. To the best of our knowledge, the variant may be the first reported deleterious synonymous variant of *MYBPC3*. Our identification enriched the spectrum of *MYBPC3* variants and emphasized the significance of synonymous variants which are always disregarded in genetic screening.

## Materials and Methods

### Subjects

The study was approved by the Institutional Review Board of Changsha Forensic Appraisal Center. Written informed consent was obtained from the legal guardian and/or next of kin of the deceased for the publication of any potentially identifiable images or data included in this article. The sample was evaluated according to postmortem measures of the International Society and Federation of Cardiology. The anatomical assessment was performed by an expert forensic pathologist and confirmed by a second independent pathologist.

### DNA/RNA Extraction and Reverse Transcription

Genomic DNA was extracted by the DNeasy Blood and Tissue Kit (Qiagen, Valencia, USA), and RNA was extracted by the RNeasy Mini Kit (Qiagen, Valencia, USA). Reverse transcription was performed using the RevertAid First Strand cDNA Synthesis Kit (Thermo, Vilnius, Lithuania).

### Whole-Exome Sequencing

Berry Genomics Company Limited (Chengdu, China) performed an exome capture, a high-throughput sequencing, and a common filtering, as described in a previous article (9). In this study, we retained all synonymous variants. After common filtering, we predicted the influence of these variants on splicing using NetGene2-2.42 (https://services.healthtech.dtu.dk/service.php?NetGene2-2.42). Causative variants were screened by the list of cardiopathy-related genes (Supplementary Table S1) and were classified based on the American College of Medical Genetics and Genomics (ACMG) Standards and Guidelines (10).

### Sanger Sequencing and Agarose gel Electrophoresis

The *MYBPC3* reference sequence and coding region (NM_000256.3) were obtained from NCBI (https://www.ncbi.nlm.nih.gov/gene/4607). Primer pairs (*MYBPC3* f: 5′-CCTCAGCTCTCTGGAATTCATC-3′, r: 5′-GGGTTTACCTTCACCTCTCATC-3′; minigene validated f: 5′-GGACTACAAGGATGACGATGAC-3′, r: 5′-CCAGCAATGACTGCGTAAGA-3′) were designed. Target fragments were amplified by polymerase chain reaction (PCR) and were analyzed using the ABI 3100 Genetic Analyzer (ABI, Foster City, USA). The PCR products were electrophoresed on 1% agarose gels.

### *MYBPC3* Minigene Model

The pcDNA3.1-MYBPC3-minigene plasmids (including exon 1, intron 1, and exon 2) were obtained from WZ Biosciences Inc. (Jinan, China). The mutagenesis of c.10G>A (pcDNA3.1-MYBPC3-10G>A) and c.24A>C (pcDNA3.1-MYBPC3-24A>C) was operated by Fast Mutagenesis Kit V2 (Vazyme, Nanjing, China).

### Hematoxylin-Eosin Staining and Immunohistochemistry Staining

Paraformaldehyde-fixed left ventricle tissue was embedded in paraffin and sliced into sections. The sections were stained with hematoxylin-eosin (HE) or immunohistochemistry of *MYBPC3* (Proteintech, 19977-1-AP, 1:200), following the protocol in our previous paper (11).

## Results

### Postmortem

**Figure 1:**
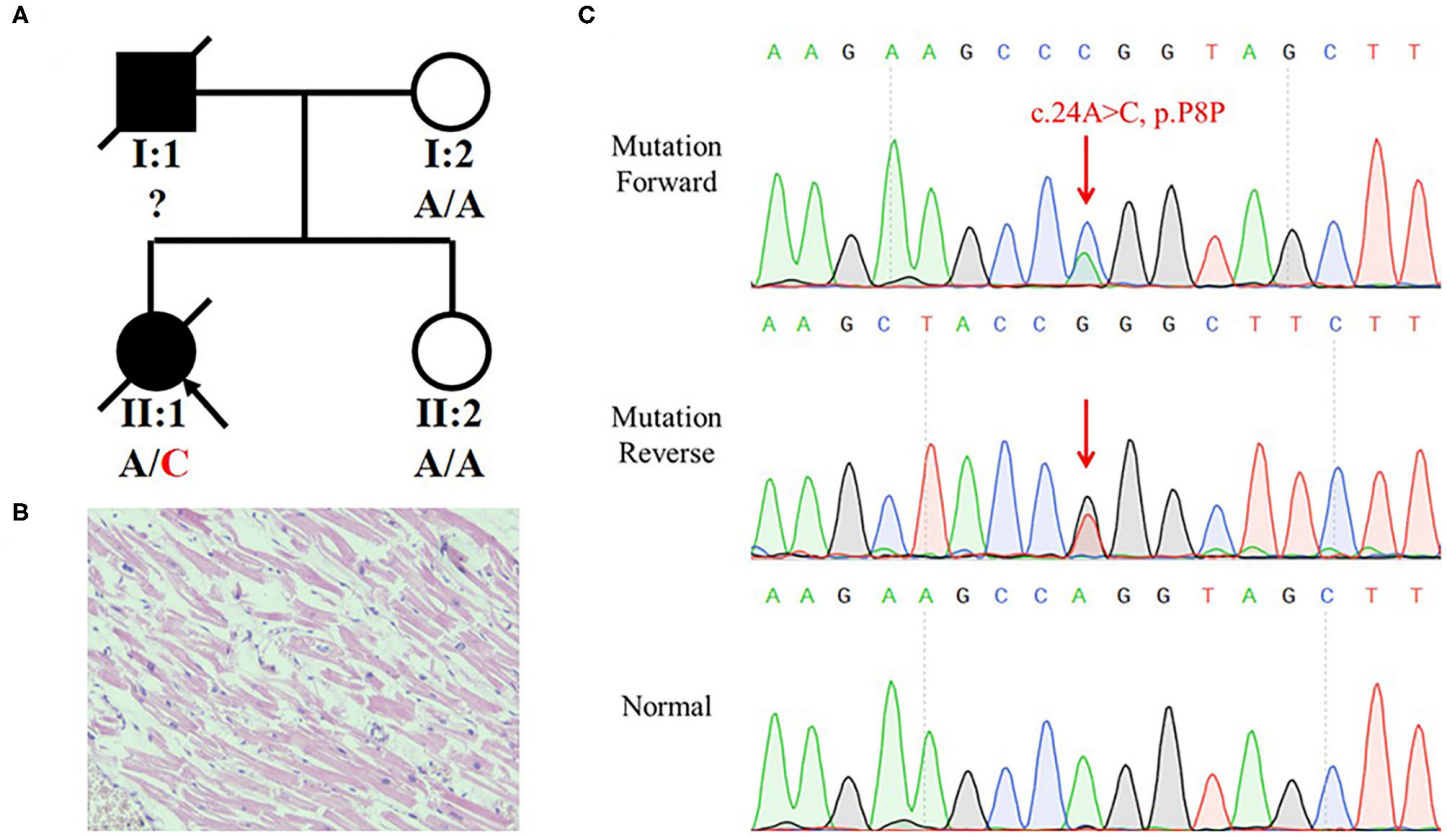
Identification of a synonymous variant of *MYBPC3* in a family with history of Sudden Cardiac Death (SCD). **(A)** Pedigree of the family with SCD. The black symbols represent the affected members and arrows indicate probands. Genotypes are identified by letters and a slash, with red representing variants. **(B)** H and E staining of left ventricle tissue of II:1 did not present hypertrophies and other anomalies. **(C)** Sequencing result of the *MYBPC3* variant (c.24A>C, p.P8P) using Sanger sequencing. Red arrow indicates the variant site.

We recruited a family with history of SCD from Central-South China (Figure 1A). The deceased (II:1) was a 29-year-old woman. At 12:00 on February 22, 2017, she expired when she sat on the sofa for a rest at home. She was diagnosed with DCM 8 years ago without other comorbidities. Pathological and toxicological tests (involving ethanol, benzodiazepines, barbitone, benzedrine, methamphetamine, ketamine, morphine, dimethylenedioxyamphetamine, and tetrahydrocannabinol acid) showed no abnormalities. Her serum IgE level was normal (27 IU/ml; reference range: 0–358 IU/ml). There was no increase of eosinophilic granulocyte (4.7%; reference range: 0.5–5%). Postmortem revealed that her heart was enlarged with valve incrassation and myocardial fibrosis, and a congestion in multiple organs with no obvious degranulation of mast cells (heart weight was 363 g; normal level: 240–260 g). The HE staining showed that cardiac muscle cells did not present the hypertrophies or other anomalies (Figure 1B). No other fatal injury or disease was observed. We concluded that she died of SCD that might have been triggered by DCM. According to a description from her mother (I:2), we speculated that her father (I:1) might have died of a heart attack at 37. The mother and sister (II:2) were unaffected.

### Genetic Analysis

The mother hoped to know whether her other daughter had a risk of SCD and turned to us for help. Blood from the heart of the deceased was collected and stored in a refrigerator at −80°C. We extracted a DNA from the blood and preformed a whole-exome sequencing (WES). We eliminated common variants using Genome Aggregation Database (GnomAD; http://gnomad.broadinstitule.org) and Chinese Millionome Database (CMDB; http://cmdb.bgi.com/cmdb/) and predicted the pathogenicity of variants using MutationTaster (http://www.mutationtaster.org/), Polyphen-2 (http://genetics.bwh.harvard.edu/pph2/), and SIFT (http://provean.jcvi.org/index.php). Five variants in cardiopathy-related genes were identified in the deceased (Table 1). Based on the ACMG classification of these variants, we highly suspected that *MYBPC3* variant (NM_000256.3: c.24A>C, p.P8P) was the genetic etiology of the deceased. The variant was classified as “Likely pathogenic” following the ACMG evidence PS3, PM2, PP1, and PP3. Although the variant was synonymous, it occurred in the penultimate base of exon 1, which may affect the RNA splicing. Sanger sequencing verified the *MYBPC3* variant in the deceased (Figure 1C). Her mother and sister did not harbor this variant.

**Table 1:** Variants identified in the deceased by whole-exome sequencing (WES) in combination with the filtration of cardiopathy-related genes.

**Gene**	**Variant**	**MutationTaster**	**PolyPhen-2**	**SIFT**	**GnomAD**	**CMDB**	**OMIM clinical phenotype**	**American college of medical genetics classification**
*LDB3*	NM_007078.2: c.2131T>C, p.S711P	D	D	D	-	-	AD, Cardiomyopathy, dilated, 1C, with or without LVNC; AD, Cardiomyopathy, hypertrophic, 24; AD, Left ventricular non-compaction 3; AD, Myopathy, myofibrillar, 4.	Uncertain significance (PM2, PP3, PP5, BP5)
*MYBPC3*	NM_000256.3: c.24A>C, p.P8P	D	-	-	-	-	AD, Cardiomyopathy, dilated, 1MM; AD/AR, Cardiomyopathy, hypertrophic, 4; AD, Left ventricular non-compaction 10.	Likely pathogenic (PS3, PM2, PP1, PP3)
*BBS2*	NM_031885.3: c.422A>G, p.N141S	D	D	T	0.00014	0.00091	AR, Bardet-Biedl syndrome 2; AR, Retinitis pigmentosa 74.	Likely benign (PM2, PP3, BS4, BP5)
*EVC2*	NM_147127.4: c.2643G>C, p.Q881H	D	B	D	0.00007	-	AR, Ellis-van Creveld syndrome; AD, Weyers acrofacial dysostosis.	Likely benign (PM2, PP3, BS4, BP5)
*BBS4*	NM_033028.4: c.1548_1549del, p.I516Mfs*8	D	-	-	0.00037	-	AR, Bardet-Biedl syndrome 4.	Likely benign (PM2, PP3, BS4, BP5)

*D, disease causing; T, tolerant; B, benign; AR, recessive dominant; AD, autosomal dominant*.

### Minigene Modeling and Immunohistochemistry Staining

**Figure 2:**
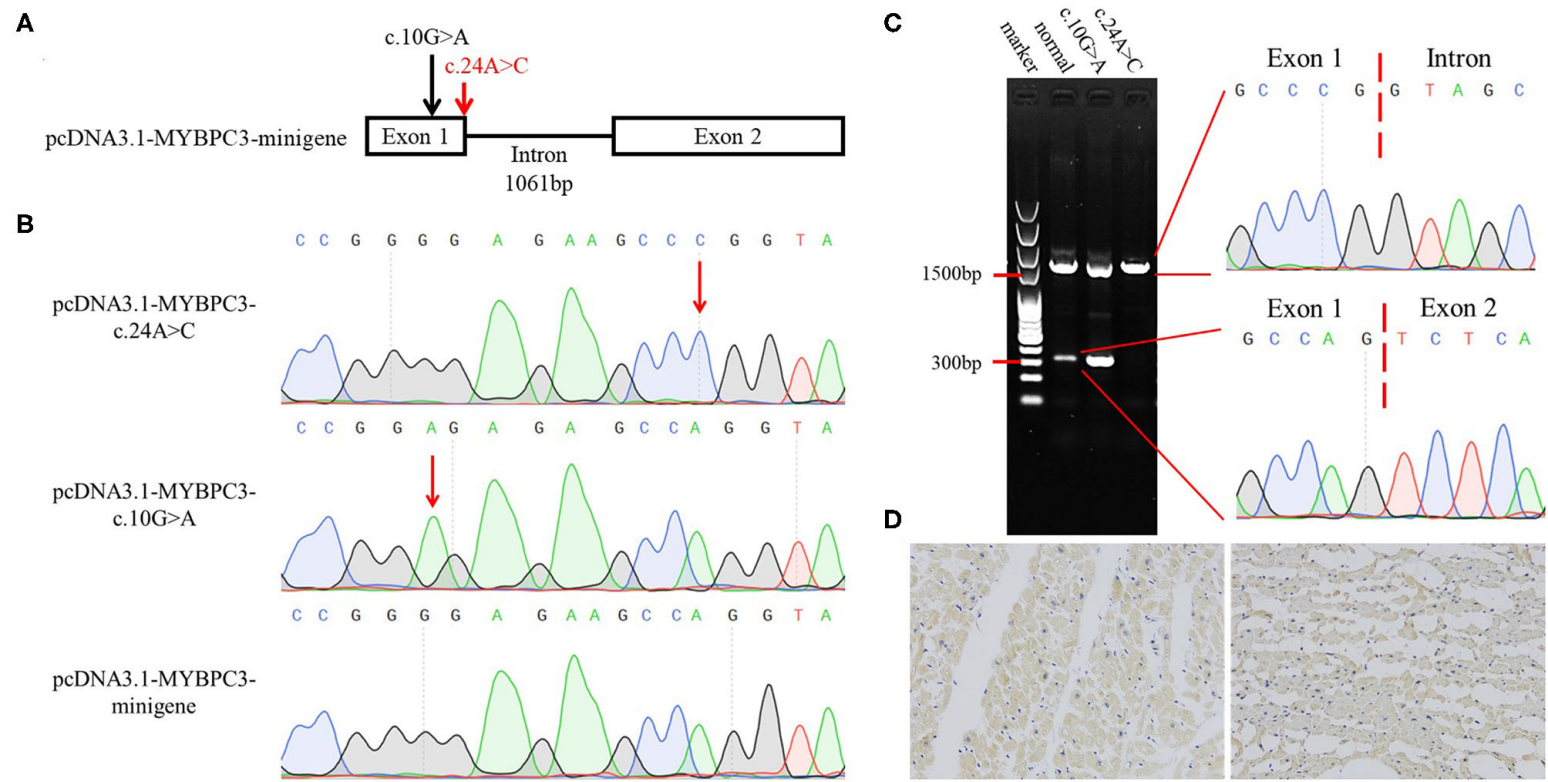
Verification of the pathogenicity of the *MYBPC3* variant (c.24A>C, p.P8P). **(A)** The schematic diagram of minigene models. Arrows indicate variant sites and red represents the variant detected in this study. **(B)** Identification of minigene models by Sanger sequencing. **(C)** Agarose gel electrophoresis and Sanger sequencing showing that c.24A>C results in the splicing alteration. **(D)** Immunohistochemistry staining of MYBPC3. Left represents the left ventricle section of the deceased with natural death, and right represents II:1.

To investigate whether the synonymous variant altered the RNA splicing, we constructed minigene models (Figure 2A). Sanger sequencing validated the mutagenesis of c.10G>A and c.24A>C in the minigene (Figure 2B). The pcDNA3.1-MYBPC3-c.10G>A plasmid was used to exclude the possibility that the mutagenesis, itself, broke the splicing site. The HEK293 cells were used in transfection. Agarose gel electrophoresis indicated that pcDNA3.1-MYBPC3-minigene model and pcDNA3.1-MYBPC3-c.10G>A model had a minigene full-length band (approximately 1,500 bp) and a spliceosome band (approximately 300 bp), while the pcDNA3.1-MYBPC3-c.24A>C model did not produce the spliceosome band, suggesting that c.24A>C caused abnormal splicing (Figure 2C). The abnormal splicing may lead to intron 1 retention and frame shift. Immunohistochemistry staining showed that compared with the deceased without SCD, the *MYBPC3* expression was decreased in the left ventricle tissue of II:1 (Figure 2D). Therefore, we inferred that the synonymous variant damaged the splicing, reduced *MYBPC3*, and was one genetic precipitating factor of her DCM and SCD.

## Discussion

Sudden cardiac death (SCD) is defined as “sudden and unexpected death occurring within an hour of the onset of symptoms, or occurring in patients found dead within 24 h of being asymptomatic, presumably due to a cardiac arrhythmia or hemodynamic catastrophe” (12). In the study, through postmortem and disease history talking, we confirmed that the deceased had DCM through negative pathological and toxicological assessment, without other fatal defects. The DCM could have triggered the hemodynamic anomaly and had threatened her life. Hence, we postulated that SCD was her cause of death. In addition, her father had a sudden death despite a momently struggle, supporting our speculation that the father also died of SCD.

Dilated cardiomyopathy (DCM) is one of the most common causes of heart failure and accounts for around 60% of childhood cardiomyopathies (6). Common causes of DCM include infection, inflammation, autoimmunity, chemical and toxin exposure, and genetic variants (13). Genetic causes are important at all ages as approximately 2% of family DCM harbor *MYBPC3* variants (6). In the study, the deceased carried a synonymous variant of *MYBPC3* (c.24A>C, p.P8P). In this variant, the penultimate base of exon 1, A, was substituted by C, which was predicted to change the splicing site. Her mother and sister, without the *MYBPC3* variant, was not found to have a DCM or other cardiovascular diseases.

According to the ACMG guideline, we listed the following evidence and determined that the synonymous variant of *MYBPC3* was “Likely pathogenic.” (1) We performed a minigene modeling and an immunohistochemistry staining and verified the damaging effect of the variant (PS3). (2) The variant was absent from controls in GnomAD and CMDB (PM2). (3) The variant was not identified in the unaffected family members (PP1). (4) MutationTaster predicted the synonymous variant to be disease-causing as a result of splice site alteration (PP4) (10). The *MYBPC3* splicing variants (c.24 + 1G>A and c.24 + 3A>C) and our variant (c.24A>C) impacted on the same splicing site. Notably, these two splicing variants were associated with HCM (14, 15). Therefore, we considered that the synonymous variant was the genetic etiology of the deceased.

To confirm the pathogenicity of our variant, we established minigene models. Generally, minigene should be constituted by the affected exon and the adjacent introns and exons at its upstream and downstream (16). In the present case, the synonymous variant was located in the first coding exon. Thus, we constructed the minigene lack of upstream sequences as it may reduce the splicing efficiency. The minigene full-length band was prominently brighter than the spliceosome band in the pcDNA3.1-MYBPC3-minigene model. In the pcDNA3.1-MYBPC3-c.24A>C model, the spliceosome band was absent, suggesting that the variant damaged the RNA splicing. We constructed the pcDNA3.1-MYBPC3-c.10G>A model to be a negative control, while variant c.10G>A promoted the splicing capacity. Mutant models exhibited the different influences of the exonic variants on the splicing and verified the pathogenicity of variant c.24A>C. The variant c.24A>C caused intron retention and frame shift, which may lead to a premature termination of translation and/or protein degradation. As per our expectation, *MYBPC3* expression was decreased in the left ventricle tissue of the deceased. Although Ito et. al. predicted and summarized the *MYBPC3* variants that alter RNA splicing, which included several missense variants, the variant (c.24A>C, p.P8P) may be the first known synonymous variant in *MYBPC3* that causes disease. This, therefore, reminded us of the significance of synonymous variants (16).

The *MYBPC3* is mapped to 11p11.2, spans more than 21 kb, and contains 35 exons (17, 18). *MYBPC3* is transversely arrayed in sarcomere A-bands, binds myosin heavy chain in thick filaments, binds titin in elastic filaments, and modulates contraction. An *MYBPC3* defect may affect the actin-myosin interactions, break sarcomere stabilities, alter calcium handling in myocardial cells, and cause DCM, HCM, or left ventricular non-compaction (8). In the study, the *MYBPC3* variant (c.24A>C, p.P8P) reduced the *MYBPC3* expression and was associated with DCM and SCD.

Sudden cardiac death (SCD) is a major public health problem worldwide. It is responsible for approximately 540,000 deaths per year in China and 170,000 to 450,000 deaths per year in the United States (12). Nearly 80% of patients with SCD had preexisting heart disease. Notably, patients presented SCD at the first clinical manifestation (19). Genetic screening of cardiopathy-related genes, such as *MYBPC3*, can contribute to assessing the risk of cardiopathy and SCD and help prevent and treat related diseases. Ehlermann et al. suggested that *MYBPC3* variants are not generally associated with a good prognosis and might cause sudden death even in asymptomatic individuals (20).

## Conclusions

In summary, we reported a young woman who died of SCD. Using WES, we identified a novel variant of *MYBPC3* (NM_000256.3: c.24A>C, p.P8P) in the deceased, which may be the first known synonymous variant of *MYBPC3* that can cause disease. It damaged the splicing and resulted in the reduced *MYBPC3* expression. Our findings expanded the genetic spectrum of SCD, confirmed the pathogenicity of the *MYBPC3* variant (c.24A>C, p.P8P), emphasized the significance of synonymous variants, and contributed to the clinical diagnosis of cardiomyopathy and SCD.

## Data Availability Statement

The original contributions presented in the study are included in the article/Supplementary Material, further inquiries can be directed to the corresponding author/s.

## Ethics Statement

Written informed consent was obtained from the deceased's legal guardian/next of kin for the publication of any potentially identifiable images or data included in this article.

## Author Contributions

J-YJ contributed to conception and design and carried out the analysis and interpretation of data. JX performed acquisition and analysis and interpretation of data. YD and YS assisted in the analysis and interpretation of data. Y-DG contributed to conception and design and wrote the original draft. RX revised the draft and approved the revisions. All authors contributed to the article and approved the submitted version.

## Funding

This work was supported by the National Science and Technology Major Project of the Ministry of Science and Technology of China (2017ZX10103005-006) and the National Natural Science Foundation of China (81970403, 82072114, and 82170598).

## Conflict of Interest

The authors declare that the research was conducted in the absence of any commercial or financial relationships that could be construed as a potential conflict of interest.

## Publisher's Note

All claims expressed in this article are solely those of the authors and do not necessarily represent those of their affiliated organizations, or those of the publisher, the editors and the reviewers. Any product that may be evaluated in this article, or claim that may be made by its manufacturer, is not guaranteed or endorsed by the publisher.
