# Supplementary material for: Case Report: Identification of the First Synonymous Variant of Myosin Binding Protein C3 (c.24A>C, p.P8P) Altering RNA Splicing in a Cardiomyopathy and Sudden Cardiac Death Case
Source: Front Cardiovasc Med. 2022 Mar 2;9:806977. doi: 10.3389/fcvm.2022.806977 (PMC8924128; doi:10.3389/fcvm.2022.806977)
Supplement: Supplementary file 1 [file Table_1.docx]

**Supplementary Table 1.** The cardiopathy-related genes list.

| *ABCC9* | *ACE* | *ACP6* | *ACTA2* | *ACTB* | *ACTC1* | *ACTN2* | *ACVR1* | *ACVR2B* | *ADRB1* |
| --- | --- | --- | --- | --- | --- | --- | --- | --- | --- |
| *ADRB2* | *ADRB3* | *AGL* | *AGT* | *AGTR1* | *AHSA2* | *ANK2* | *ANKRD1* | *APOBEC2* | *APPB1* |
| *ARL13B* | *ARL6* | *ASXL2* | *ATE1* | *ATP1A2* | *ATP4A* | *ATP4B* | *BAT1* | *BBIP1* | *BBS1* |
| *BBS10* | *BBS12* | *BBS2* | *BBS4* | *BBS5* | *BBS7* | *BBS9* | *BCL11A* | *BCL6* | *BCL9* |
| *BCOR* | *BICC1* | *BMP7* | *BMPR1A* | *BMPR1B* | *BMPR2* | *BUB1B* | *C1ORF106* | *CACNA1C* | *CACNA2D1* |
| *CALM1* | *CALM2* | *CALR3* | *CASQ2* | *CAV3* | *CCDC39* | *CCDC40* | *CCT4* | *CDH1* | *CDH2* |
| *CDK13* | *CDKN1C* | *CER1* | *CFC1* | *CHD1L* | *CHD2* | *CHD4* | *CHD7* | *CHRAC1* | *CHRD* |
| *CHRNG* | *CITED2* | *CLDN7* | *CLUL1* | *CNTF* | *COL11A1* | *COL11A2* | *COL2A1* | *COL3A1* | *CREBBP* |
| *CRELD1* | *CRHBP* | *CRX* | *CRYAB* | *CSRP1* | *CSRP3* | *CTLA4* | *CTNNA3* | *CUL3* | *CYP11B2* |
| *DAND5* | *DAPK3* | *DES* | *DHCR24* | *DHCR7* | *DHODH* | *DLL1* | *DMRT2* | *DNAI1* | *DNAI2* |
| *DOLK* | *DOT1L* | *DPP6* | *DPPA4* | *DSC2* | *DSG2* | *DSP* | *DST* | *DTNA* | *DVL1* |
| *DVL2* | *DZIP1* | *EDNRA* | *EDNRB* | *EED* | *EFNB1* | *EHMT1* | *ELN* | *EMD* | *EP300* |
| *ESCO2* | *EVC* | *EVC2* | *EYA4* | *EZH1* | *EZH2* | *FBN1* | *FBN2* | *FGB* | *FH19* |
| *FIBP* | *FKTN* | *FLNA* | *FLNB* | *FMO5* | *FOXA2* | *FOXC1* | *FOXC2* | *FOXH1* | *FOXJ1* |
| *FOXL2* | *FTO* | *FXN* | *GAA* | *GADL1* | *GALNT11* | *GATA4* | *GATA5* | *GATA6* | *GATAD1* |
| *GDF1* | *GJA1* | *GJA5* | *GJA8* | *GJA9* | *GLA* | *GLI2* | *GLI3* | *GPC3* | *GPD1* |
| *GPD1L* | *GPR161* | *GPRC6A* | *GRID2* | *GSK3B* | *HAND1* | *HAND2* | *HCN4* | *HES1* | *HES4* |
| *HEY2* | *HFE* | *HMGCL* | *HOXA1* | *HUWE1* | *HYLS1* | *ID2* | *IDUA* | *IER2* | *IFNG* |
| *IFT122* | *IFT172* | *IFT20* | *IFT57* | *IFT88* | *IGFBP4* | *IGFBP5* | *IHH* | *IL10* | *IPPK* |
| *ISL1* | *JAG1* | *JARID2* | *JAZF1* | *JPH2* | *JUP* | *KAT6B* | *KCND2* | *KCND3* | *KCNE1* |
| *KCNE1L* | *KCNE2* | *KCNE3* | *KCNE4* | *KCNH2* | *KCNJ11* | *KCNJ2* | *KCNJ5* | *KCNJ8* | *KCNMB1* |
| *KCNQ1* | *KCNT1* | *KDM5A* | *KDM5B* | *KDM6A* | *KIAA0196* | *KIAA1841* | *KIF3A* | *KIF3B* | *KIF3C* |
| *KIFAP3* | *KLF13* | *KMT2D* | *KRAS* | *LAMA2* | *LAMA4* | *LAMP2* | *LBR* | *LDB3* | *LEFTY1* |
| *LEFTY2* | *LEMD3* | *LIPC* | *LLPH* | *LMNA* | *LPIN1* | *LRRC50* | *LRRC6* | *MARK2* | *MAX* |
| *MED13L* | *MED20* | *MEF2A* | *MEF2C* | *MESP1* | *METT10D* | *MGAT1* | *MGP* | *MICA* | *MICB* |
| *MID1* | *MKKS* | *MKRN2* | *MKS1* | *MNDA* | *MSX2* | *MYBPC3* | *MYH10* | *MYH11* | *MYH6* |
| *MYH7* | *MYH7B* | *MYL2* | *MYL3* | *MYLK2* | *MYOF* | *MYOZ2* | *MYPN* | *MYRF* | *NAA15* |
| *NCOA6* | *NCOR2* | *NEBL* | *NEK2* | *NELFA* | *NEXN* | *NF1* | *NFATC1* | *NFATC3* | *NFATC4* |
| *NFKBIL1* | *NGF* | *NIPBL* | *NKD1* | *NKX2-5* | *NKX2-6* | *NKX3-2* | *NODAL* | *NOS3* | *NOTCH1* |
| *NOTCH2* | *NOTCH2NL* | *NOTCH3* | *NOTCH4* | *NOTO* | *NPHP3* | *NPPA* | *NPPB* | *NSD1* | *NUB1* |
| *NUMBL* | *NUP188* | *OBSCN* | *OFD1* | *OSR1* | *PAFAH1B1* | *PAPOLG* | *PCMTD2* | *PCSK5* | *PDLIM3* |
| *PEX1* | *PEX13* | *PHF8* | *PHYHD1* | *PIFO* | *PITX2* | *PKD1L1* | *PKD2* | *PKP2* | *PLA2G7* |
| *PLAGL1* | *PLEC* | *PLN* | *PPM1K* | *PPP3CA* | *PQBP1* | *PRC1* | *PRDM1* | *PRKAB2* | *PRKAG2* |
| *PRKD1* | *PROX1* | *PSEN1* | *PSEN2* | *PTCH1* | *PTCH2* | *PTPLA* | *PTPN11* | *PTPN22* | *PTPRC* |
| *RAB10* | *RAB23* | *RAF1* | *RAI1* | *RAI2* | *RANGRF* | *RAPGEF5* | *RBM20* | *RECQL5* | *REL* |
| *RFX2* | *RFX3* | *RIT1* | *RNF20* | *RNF207* | *ROCK2* | *ROR2* | *RPGRIP1L* | *RPSA* | *RUNX2* |
| *S100Z* | *SALL1* | *SALL2* | *SALL4* | *SATB2* | *SCN1B* | *SCN3B* | *SCN4B* | *SCN5A* | *SCNN1D* |
| *SDC2* | *SDHA* | *SEL1L3* | *SEMA3E* | *SESN1* | *SETBP1* | *SGCA* | *SGCB* | *SGCD* | *SGCE* |
| *SGCG* | *SHH* | *SHOC2* | *SIX3* | *SLC26A2* | *SLC2A10* | *SLMAP* | *SMAD2* | *SMAD5* | *SMARCA4* |
| *SMARCD3* | *SMO* | *SMYD1* | *SMYD2* | *SNAI1* | *SNTA1* | *SOD2* | *SOS1* | *SOX17* | *SOX9* |
| *SRF* | *STIL* | *SUFU* | *SUPT3H* | *SUPT5H* | *SUV420H1* | *TAZ* | *TBX1* | *TBX20* | *TBX3* |
| *TBX5* | *TCAP* | *TCF21* | *TCOF1* | *TDGF1* | *TFAP2A* | *TFAP2B* | *TGFB1* | *TGFBR1* | *TGFBR2* |
| *TGIF1* | *TLL1* | *TMBIM4* | *TMEM195* | *TMEM43* | *TMPO* | *TNF* | *TNFRSF21* | *TNNC1* | *TNNI3* |
| *TNNT2* | *TP63* | *TPM1* | *TRDN* | *TRIM32* | *TRPM4* | *TSC1* | *TSEN15* | *TTC21B* | *TTC30A* |
| *TTC8* | *TTN* | *TTR* | *TWIST1* | *TXNDC3* | *UBE2B* | *UBR1* | *UMODL1* | *USF1* | *USP34* |
| *USP44* | *VANGL2* | *VCL* | *VEGFA* | *VEGFC* | *VIT* | *WDR5* | *WHSC1* | *WNT3A* | *XPO1* |
| *ZEB2* | *ZFPM1* | *ZIC3* | *ZNF480* | *ZNF528* | *ZNF534* | *ZNF610* | *ZNF638* | *ZNHIT3* |  |
